# Case Report: The lost art of cisterna magna puncture—teaching video

**DOI:** 10.3389/fsurg.2025.1595249

**Published:** 2025-09-26

**Authors:** Viktor Weiss, Michal Harsany, Jaroslav Brichta, Martin Kojan, Roman Herzig, Vladimír Cervenak, Pavel Filip, Petr Aulicky

**Affiliations:** 1 Department of Neurology, Faculty of Medine, University Hospital of St. Anne and Masaryk University, Brno, Czechia; 2 International Clinical Research Centre, St. Anne’s University Hospital in Brno, Brno, Czechia; 3 Department of Neurology, Charles University Faculty of Medicine, Hradec Králové, Czechia; 4 Department of Neurology, Comprehensive Stroke Center, University Hospital Hradec Králové, Hradec Králové, Czechia; 5 Department of Radiology, Faculty of Medicine, St. Anne’s University Hospital, Masaryk University, Brno, Czechia; 6 Department of Neurology, Charles University, First Faculty of Medicine and General University Hospital, Prague, Czechia; 7 Center for Magnetic Resonance Research (CMRR), University of Minnesota, Minneapolis, MN, United States; 8 Department of Cybernetics, Czech Technical University in Prague, Prague, Czechia; 9 Hospital of the Brothers of Charity Brno, Brno, Czechia

**Keywords:** cisternal puncture, CSF, intracranial hypertension, case report, teaching video

## Abstract

The early 20th century saw the emergence and fall of cisterna magna puncture as a therapeutic and diagnostic tool, purportedly offering distinct advantages over lumbar puncture. However, the renewed interest in this procedure as a potential access point in intrathecal gene therapy indicates that it may not be completely forgotten. In light of the limited reports and the virtual loss of practical expertise with this procedure among specialised clinicians, this instructional video is offered as a valuable resource for clinicians or researchers not only in diagnostic settings, but also as an access to the central distribution point of cerebrospinal fluid with therapeutical intents in the future, as envisaged by medical pioneers a century ago.

## Introduction

The cisterna magna or suboccipital puncture, now seemingly antiquated, is a procedure originally developed in the early 20th century to obtain cerebrospinal fluid (CSF) for both diagnostic and therapeutic purposes. Largely superseded by lumbar puncture due to reliability and substantially lower risk of catastrophic adverse events, it has recently seen renaissance as an envisioned method for the administration of advanced therapeutic agents in a range of congenital and acquired conditions (1, 2). Moreover, the suboccipital approach remains a viable alternative for the acquisition of diagnostic CSF samples in instances where lumbar puncture is anatomically unfeasible or contraindicated.

Nonetheless, the very concept of inserting a needle into the close proximity of vitally important centres in the medulla oblongata elicited substantial uneasiness in clinicians from the inception of the technique. Indeed, its pioneers, Obregia (3), Ayer (4) and Eskuchen (5) strongly advocated for extreme caution, “steady hand and a stout heart” (6), even to the point of recommending previous experience in cadavers prior to performing the procedure clinically (4, 7). On the other hand, early reports of hundreds of successful cases with seemingly minimal rate of adverse events (8) were enthusiastic and presented a large range of diagnostic and therapeutic opportunities, ranging from localisation of obstructive lesions of spinal cord to the intrathecal administration of anti-meningococcal serum, or salvarsanised serum in neurosyphilis (6). The early proponents of cisterna magna puncture for serum therapy regarded cisterna magna as a conduit which was, contrary to lumbar approach, capable of facilitating an effective dissemination of therapeutic agents throughout the central nervous system, as demonstrated by intrathecal ink experiments. However, the rise of sporadic reports of severe adverse events such as subarachnoid haemorrhage, cessation of respiration and death in the literature, combined with the revolution of the treatment of infectious diseases by the discovery of penicillin and progress in the diagnostic capabilities of non-invasive imaging modalities, meant a general shift in the risk-benefit ratio of the procedure until it was virtually abandoned.

**Figure 1:**
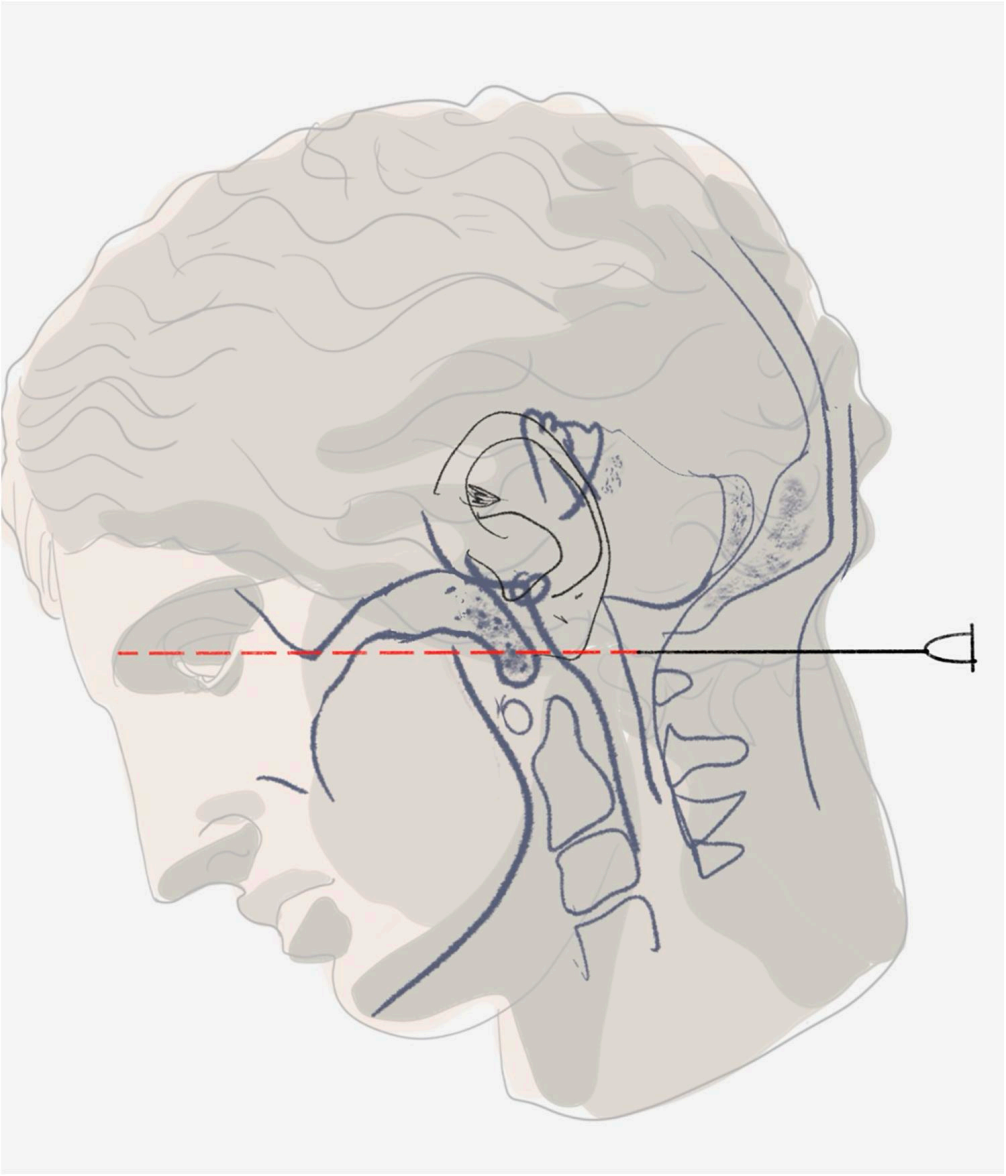
Schematic of midsagittal brain section with the needle *in situ* reaching into the cisterna magna, marking glabella and the external auditory meatus as anatomical landmarks for the plane of needle insertion.

Several approaches have been developed over the years. The method of choice in the USA was Ayer's technique (4): with the patient positioned in lateral decubitus and slight cervical flexion, the needle was introduced at the midline of the posterior suboccipital region, just superior to the spine of the axis, and advanced with caution along a plane aligned with the glabella and the external auditory meatus (see Figure 1). The posterior occipito-atlantoid ligament was penetrated at the average depth of 4–6.5 cm, followed by the dura mater. In the subsequent step, the needle was advanced an additional 1–2 mm and the stylet removed to evaluate for CSF outflow. In its absence, the needle was gradually advanced until the desired flow was obtained or a depth too disproportionate was reached, with 8 cm proposed by other authors (8). A sudden loss of resistance could also be used as an indication of the entry of the needle into the cisterna magna. Eskuchen's technique (5), advocated by the German medical community, directed the needle obliquely towards the posterior margin of the foramen magnum. Upon contact with the occipital bone, the needle was slightly withdrawn and advanced at a slightly lowered angle to enter the cisterna magna. And lastly, lateral technique was proposed as an alternative to the previous midline approaches (9). With the patient in supine position, the needle is inserted 1 cm inferior and 1 cm posterior to the apex of the mastoid process, specifically between the lower margin of the occipital bone and the atlas, to the expected depth in adults of about 5.5–6.5 cm.

In addition to the guidelines proposed a century ago, the development of modern medicine and risk of the presence of iatrogenic coagulopathy call for further preparatory measures, including the following:

- A thorough review of patient's current medication regimen, with particular attention to anticoagulation and/or antiplatelet agents, which should be discontinued in accordance to established guidelines for analogous procedures
- An assessment of the patient's history of allergies to contrast agents, local sterilisation products and eventual anaesthetic agents, if to be utilised
- Head imaging to exclude abnormities at the cervico-occipital junction and skull base, accompanied by computed tomography (CT) and/or magnetic resonance (MRI) angiography to visualise the vertebral arteries, including the V3 segment, or eventual vascular anomalies crossing the spinal canal at the C1-C2 puncture site
- Local sterilisation of puncture site prior to the start of the procedure
- Local infiltration anaesthesia, as recommended by the proponents of the lateral approach (9) may also be considered.

## Case report

In this account, we present a 42-year-old male, previously healthy patient who gradually, over the duration of six months following the initial, possibly related symptom of neck stiffness, developed progressive, non-specific complaints. These ranged from subjective vertigo in reclined head position, intermittent tension headache accompanied by blurry vision, to several episodes of nausea with vomiting. Upon the admission to the First Department of Neurology at the University Hospital of St. Anne, the clinical neurological examination revealed slower cognitive processing speed, attention deficit, and impairments in working and short-term episodic memory. Fundoscopic examination indicated substantially elevated intracranial pressure, evidenced by pronounced macular oedema.

**Figure 2:**
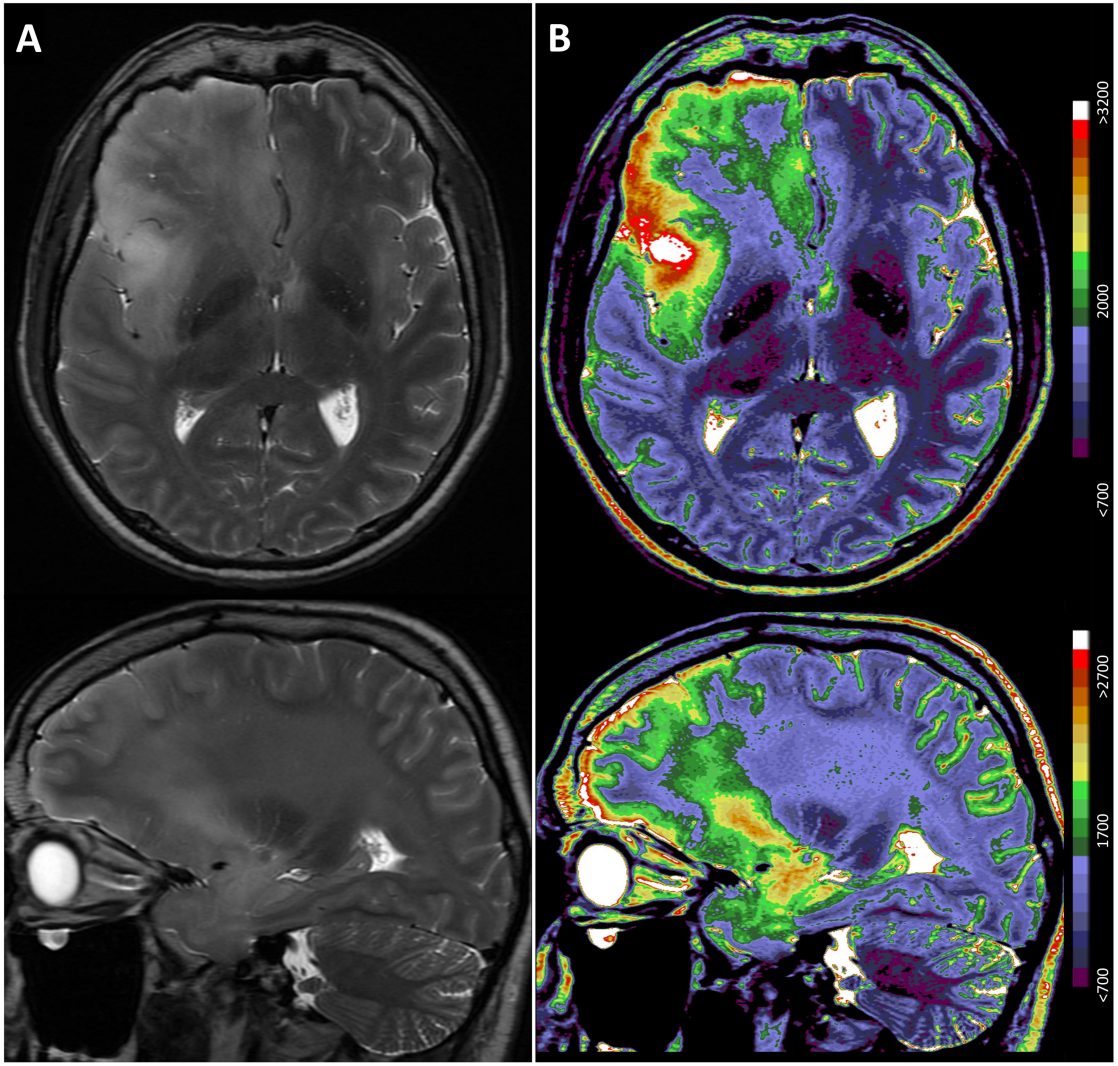
MRI T2-weighted scan (transversal and sagittal sections) of the presented patient, showing expansive lesion in the right-side medial frontal and temporal lobe with a massive oedema, affecting also corpus callosum and introducing midline shift, and a contrast-enhanced suprasellar expansion compressing the third ventricle and dislocating the optic chiasm. **(A)** Standard grayscale. **(B)** Complex colour scale for better visualisation, including the colour coding of T2-weighted values.

An MRI scan of brain was performed, revealing an expansive lesion in the right medial frontal and temporal lobes, accompanied by massive oedema affecting also corpus callosum and introducing midline shift. Additionally, a contrast-enhanced suprasellar mass was observed, compressing the third ventricle and displacing the optic chiasm (see Figure 2). No elevation of choline/N-acetylaspartate ratio was detected in the lesions in a follow-up magnetic resonance spectroscopy. Electroencephalography findings were unremarkable, and serological tests including tests for human immunodeficiency virus, herpes simplex virus, varicella-zoster virus, Epstein–Barr virus, cytomegalovirus, tick-borne encephalitis, Mycobacterium tuberculosis, Toxoplasma gondii, and other tissue parasites failed to yield any aetiologically conclusive results. Additional antibody tests to rule out autoimmune encephalitis along with paraneoplastic antibody screening were also negative. Chest x-ray scan, followed by CT scans of the chest and abdomen, and abdominal ultrasound revealed normal findings, with the exception of asymptomatic nephrolithiasis.

Cerebral oedema therapy consisting of intravenous dexamethasone and mannitol was initiated, combined with cefazoline to address a potential infectious aetiology. Mild hyponatraemia suggestive of syndrome of inappropriate antidiuretic hormone secretion was managed using sodium chloride and fluid intake restriction. Additional therapeutic interventions included analgesic agents (tramadol, paracetamol, metamizole), low-molecular weight heparin (enoxaparin), parahypopituitarism management (levothyroxine, hydrocortisone), antidepressant and anxiolytic agents (citalopram, alprazolam), and antihypertensive agent (pantoprazole).

An interdisciplinary committee comprising neurologists, oncologists, neurosurgeons and imaging specialists convened to evaluate several principal avenues for further investigation, including tumour biopsy and CSF analysis. In light of the clinical and radiological evidence indicating markedly elevated intracranial pressure, there was broad consensus that both tumour biopsy and direct ventricular access for CSF sampling would entail an unacceptably high risk of tonsillar herniation. Although it was acknowledged that both lumbar puncture and cisterna magna puncture are inherently hazardous under such conditions, the committee ultimately deemed access via the cisterna magna to be the safer option under the presented condition. Following a thorough and extensive discussion with the patient, cisterna magna puncture using Ayer's technique was selected (see Supplementary Video 1). A complex monitoring protocol was employed in the intensive care unit under the supervision of an experienced anaesthesiologist, including the continuous assessment of blood pressure, heart rate, and oxygen saturation. Analgosedation was administered using intravenous midazolam (2 mg), sufentanil (5 μg), and 1% propofol (fractionated total dose of 60 mg). A PAJUNK 20 G × 120 mm lumbar needle was selected for the procedure, which was completed without complications. The analysis of the collected CSF sample revealed no evidence of infection. MYD88 gene test in CSF was negative and no peripheral signs of lymphoma were detected—flow cytometry of peripheral blood and blood marrow were unremarkable.

An open biopsy of the expansive fronto-parietal lesion based on guided craniotomy was performed, and histological examination confirmed a diagnosis of grade 4 astrocytoma, with the presence of IDH1 R132H mutation. Surgical resection was not deemed feasible due to the extent of the pathology. Adjuvant radiotherapy, continuous physiotherapy, gradual discontinuation of corticosteroid therapy, and ongoing management of endocrine dysfunction were recommended.

At the discharge, the patient was in a stable cardio-pulmonary condition, with mild signs of prefrontal syndrome. However, approximately six months later, the patient succumbed to complications associated with the primary diagnosis as described above.

## Conclusion

While not devoid of the risk of substantial complications, cisterna magna puncture may offer a viable diagnostic option in complicated cases such as the patient outlined above. Furthermore, the advent of novel genetic therapeutic approaches has positioned this procedure as a promising new direction for intrathecal administration of viral vectors due to its markedly enhanced effectivity in gene transfer to the brain tissues than the conventional lumbar puncture. Given the scarcity of reports and virtual loss of practical knowledge and experience with this procedure among neurologists and neurosurgeons, this teaching video could provide a valuable stepping stone for clinicians or researchers considering the reintroduction of this procedure as an access to this central distribution point of CSF envisioned by medical pioneers a century ago. It will be for the future researchers to ascertain whether the potential benefits of intracisternal gene therapy can outweigh the associated risks.

## Data Availability

The datasets presented in this article are not readily available because of ethical and privacy restrictions. Requests to access the datasets should be directed to the corresponding author.

## Supplementary material

Supplementary Video 1 Cisterna magna puncture, performed by VW.
